# Teicoplanin-based antimicrobial therapy in *Staphylococcus aureus* bone and joint infection: tolerance, efficacy and experience with subcutaneous administration

**DOI:** 10.1186/s12879-016-1955-7

**Published:** 2016-11-03

**Authors:** Olivier Peeters, Tristan Ferry, Florence Ader, André Boibieux, Evelyne Braun, Anissa Bouaziz, Judith Karsenty, Emmanuel Forestier, Frédéric Laurent, Sébastien Lustig, Christian Chidiac, Florent Valour, on behalf of the Lyon BJI study group, Tristan Ferry, Florent Valour, Thomas Perpoint, André Boibieux, François Biron, Patrick Miailhes, Florence Ader, Julien Saison, Sandrine Roux, Claire Philit, Fatiha Daoud, Johanna Lippman, Evelyne Braun, Christian Chidiac, Yves Gillet, Laure Hees, Sébastien Lustig, Philippe Neyret, Olivier Reynaud, Adrien Peltier, Olivier Cantin, Michel-Henry Fessy, Anthony Viste, Philippe Chaudier, Romain Desmarchelier, Thibault Vermersch, Sébastien Martres, Franck Trouillet, Cédric Barrey, Francesco Signorelli, Emmanuel Jouanneau, Timothée Jacquesson, Ali Mojallal, Fabien Boucher, Hristo Shipkov, Mehdi Ismail, Joseph Chateau, Frederic Laurent, François Vandenesch, Jean-Philippe Rasigade, Céline Dupieux, Isabelle Morelec, Marc Janier, Francesco Giammarile, Michel Tod, Marie-Claude Gagnieu, Sylvain Goutelle, Eugénie Mabrut

**Affiliations:** 1 Regional Referral Center for Bone and Joint Infection, Hospices Civils de Lyon, Lyon, France; 2 Infectious Disease Department, Hospices Civils de Lyon, Groupement Hospitalier Nord, 103 Grande Rue de la Croix-Rousse, 69004 Lyon, France; 3 Department of General Medicine, Claude Bernard Lyon 1 University, Lyon, France; 4 INSERM U1111, International Center for Research in Infectiology (CIRI), Claude Bernard Lyon 1 University, Lyon, France; 5 Department of Infectious Diseases, Lucien Hussel Hospital Center, Vienne, France; 6 Department of Infectious Diseases, William Morey Hospital Center, Châlon-sur-Saône, France; 7 Department of Infectious Diseases, Centre Hospitalier Métropole Savoie, Chambéry, France; 8 Laboratory of Bacteriology, National Reference Center for Staphylococci, Groupement Hospitalier Nord, Hôpital de la Croix-Rousse, Hospices Civils de Lyon, Lyon, France; 9 Department of Orthopedic Surgery, Groupement Hospitalier Nord, Hôpital de la Croix-Rousse, Hospices Civils de Lyon, Lyon, France

**Keywords:** Bone and joint infection, *Staphylococcus aureus*, Teicoplanin, Subcutanous administration

## Abstract

**Background:**

*Staphylococci* represent the first etiologic agents of bone and joint infection (BJI), leading glycopeptides use, especially in case of methicillin-resistance or betalactam intolerance. Teicoplanin may represent an alternative to vancomycin because of its acceptable bone penetration and possible subcutaneous administration.

**Methods:**

Adults receiving teicoplanin for *S. aureus* BJI were included in a retrospective cohort study investigating intravenous or subcutaneous teicoplanin safety and pharmacokinetics.

**Results:**

Sixty-five *S. aureus* BJIs (orthopedic device-related infections, 69 %; methicillin-resistance, 17 %) were treated by teicoplanin at the initial dose of 5.7 mg/kg/day (IQR, 4.7–6.5) after a loading dose of 5 injections 12 h apart. The first trough teicoplanin level (C_min_) reached the therapeutic target (15 mg/L) in 26 % of patients, only. An overdose (C_min_ >25 mg/L) was observed in 16 % patients, 50 % of which had chronic renal failure (*p* = 0.049). Seven adverse events occurred in 6 patients (10 %); no predictive factor could be highlighted. After a 91-week follow-up (IQR, 51–183), 27 treatment failures were observed (42 %), associated with diabetes (OR, 5.1; *p* = 0.057), systemic inflammatory disease (OR, 5.6; *p* = 0.043), and abscess (OR, 4.1; *p* < 10^−3^). A normal CRP-value at 1 month was protective (OR, 0.2; *p* = 0.029). Subcutaneous administration (*n* = 14) showed no difference in pharmacokinetics and tolerance compared to the intravenous route.

**Conclusions:**

Teicoplanin constitutes a well-tolerated therapeutic alternative in *S. aureus* BJI, with a possible subcutaneous administration in outpatients. The loading dose might be increase to 9–12 mg/kg to quickly reach the therapeutic target, but tolerance of such higher doses remains to be evaluated, especially if using the subcutaneous route.

**Electronic supplementary material:**

The online version of this article (doi:10.1186/s12879-016-1955-7) contains supplementary material, which is available to authorized users.

## Background

Staphylococci are the first etiologic agents of bone and joint infection (BJI). Methicillin-susceptible *Staphylococcus aureus* (MSSA) is predominant and antistaphylococcal penicillins such as nafcillin, oxacillin and cloxacillin are the backbone molecules for the initial antimicrobial therapy [1, 2]. Nevertheless, glycopeptide alternative, including vancomycin or teicoplanin, remains frequently used for several reasons: i) although hospital diffusion of methicillin-resistant clones of *S. aureus* (MRSA) is currently controlled in France, MRSA still accounts for 20 % of *S. aureus* isolates involved in BJI [3]; ii) half of staphylococcal BJI are caused by coagulase negative staphylococci (CNS), among which methicillin resistance has continuously increased in the past years to presently reach 50 % of isolates [3]; and iii) antistaphylococcal penicillins are the first cause of antimicrobial-related adverse events during long-term treatment of staphylococcal BJI [4], in case of which glycopeptides are the first alternative. If vancomycin is largely prescribed in this context, teicoplanin could theoretically represent an acceptable alternative in BJI as studies have shown a comparable efficacy compared to vancomycin in various other conditions [5] and an improved safety profile with fewer renal toxicity [6], as well as the possibility of daily subcutaneous injection, of particular interest for outpatient parenteral antimicrobial therapy (OPAT). In addition, various studies have shown that teicoplanin pharmacodynamic profile was superior compared to vancomycin regarding bone diffusion [7, 8]. Few studies have investigated the use of teicoplanin in BJI, particularly through subcutaneous administration. The present study assesses the efficacy and tolerance of teicoplanin in *S. aureus* BJI, especially focusing on subcutaneous use.

## Methods

### Inclusion criteria and data collection

A retrospective single-center observational cohort study (2001 to 2011) was conducted including all consecutive patients managed at our institution receiving teicoplanin as part of *S. aureus* BJI treatment. Patients diagnosed with staphylococcal BJI were identified by cross-referencing the prospective maintained databases of the regional referral center for BJI and the bacteriology laboratory, which list exhaustively all strains isolated from osteoarticular samples since 2001. Patients with diabetic foot- and decubitus ulcer-related BJI were excluded, as they require a specific management [9]. If patients presented more than one osteoarticular infected site, they were considered as independent events for cohort description and outcome analysis, but pooled for tolerance and pharmacologic evaluation. For each patient, data were collected from medical records, nursing charts and biological software in an anonymous standardized case report form. All available trough teicoplanin plasmatic levels (C_min_) in the first 14 days of treatment were recorded.

### Definitions

BJI diagnosis was based upon the existence of clinical and biological evidences of infection, and at least one reliable bacteriological sample positive for *S. aureus* (i.e., percutaneous joint fluid aspiration, surgical sample, and/or blood culture). BJI were classified according to: i) the existence of orthopedic implant (i.e. joint prosthesis, peripheral or vertebral osteosynthesis); and ii) progression of infection, differentiating acute (≤3 weeks) *versus* chronic (>3 weeks) infection, calculated from the presumed date of inoculation (i.e., date of device implantation for postoperative orthopaedic device-related infection (ODI), or date of symptom onset for native BJI) up to diagnosis.

The modified Charlson’s comorbidity index was calculated as previously described [10]. Immunosuppression was defined as: i) corticosteroid therapy >10 mg of prednisone per day or equivalent for at least 3 months; ii) immunosuppressive drug(s) during the two last months before BJI onset; or iii) chemotherapy for hematological malignancy or solid tumor.

A C_min_ >15 mg/L was taken as an acceptable therapeutic target. Patients with a C_min_ >25 mg/L were considered as overexposure.

Teicoplanin-related adverse events (AE) occurring during follow-up were notified and classified according to the Common Terminology Criteria for Adverse Events (CTCAE, National Cancer Institute, 2003). Teicoplanin accountability in the AE occurrence was left to the clinician appreciation, with the help of a pharmacovigilance specialist in doubtful cases.

Treatment failure was defined as persisting infection under appropriate antimicrobial therapy, relapse after the interruption of antimicrobial therapy, necessity of surgical revision on the account of persisting septic focus ≥5 days after the first intervention, superinfections, and/or fatal outcome if BJI-related.

### Teicoplanin administration

For intravenous (IV) administration, each dose was diluted in 100 mL of isotonic saline solution and administrated over a 30-min period. For subcutaneous (SC) injections, each dose was diluted in 50 mL of isotonic saline solution and delivered by a nurse during a 30- to 60-min gravity infusion using a butterfly disposable needle.

### Statistical analysis

Descriptive statistics were used to estimate the frequencies of the study variables, described as percentages (%) for dichotomous variables, and medians (interquartile range (IQR)) for continuous variables. For the percentage calculation of each variable, the number of missing values was excluded from the denominator. Non-parametric statistical methods were used to compare the study groups (Chi-squared test, Fisher exact test and Mann–Whitney *U* test), as appropriate. Determinants of teicoplanin-related AE and treatment failure were assessed using binary logistic regression, including the clinically relevant variables in each model, and expressed by their Odd ratio (OR) and 95 % confidence intervals (95 % CI). Clinically pertinent variables with a *p*-value <0.15 in the univariate analysis were included in the final multivariate models. A value of *p* <0.05 was considered as significant. All analyses were performed using SPSS software version 19.0 (SPSS, Chicago, IL).

## Results

### Population characteristics

Sixty patients were included (34 male, 56.7 %; median age, 62 years (interquartile range (IQR), 48-75), among who two and one presented three and two concomitant infected osteoarticular site, respectively. Consequently, a total of 65 episodes of BJI were analyzed, including 20 (30.8 %) native and 45 (69.2 %) orthopedic device-related (ODI) infections, and 23 (35.4 %) chronic infections. A MRSA was implicated in 11 (16.9 %) cases and 17 (26.2 %) infections were plurimicrobial. All staphylococcal isolates were susceptible to teicoplanin. A surgical procedure was performed in 50 (76.9 %) cases, predominantly in ODI (93.3 %). All patients were initially treated by a combination antimicrobial therapy. Median total duration of treatment was 26.8 (IQR, 17.7–42.8) weeks. Patients’ characteristics are described in Table 1.

**Table 1:** Description of the 65 included episodes of BJI and comparison between the intravenous and subcutaneous routes of administration

	Total population (*n* = 65)	Intravenous administration (*n* = 51)	Subcutaneous administration (*n* = 14)	*p*-value
Demographics				
Sex (male)	34 (52.3 %)	27 (52.9 %)	7 (50.0 %)	1.000
Age (year-old)	61.8 (49.0–74.0)	61.8 (52.1–73.8)	59.1 (39.1–75.5)	0.678
Comorbidities				
Modified CCI	3 (1–5)	3 (2–5)	1.5 (0.3–5.8)	0.478
BMI (kg/m^2^)	27.0 (21.6–29.7)	27.8 (22.0–31.6)	24.7 (20.7–28.0)	0.154
Obesity (BMI > 30)	14 (22.2 %)	13 (26.5 %)	1 (7.1 %)	0.116
Diabetes	8 (12.3 %)	7 (13.7 %)	1 (7.1 %)	0.447
Immunosuppression	11 (16.9 %)	10 (19.6 %)	1 (7.1 %)	0.253
Chronic renal failure	9 (14.8 %)	6 (12.8 %)	3 (21.4 %)	0.338
Chronic hepatic disease	2 (3.3 %)	2 (4.3 %)	0 (0 %)	0.591
Chronic pulmonary disease	15 (24.6 %)	10 (21.3 %)	5 (35.7 %)	0.223
Congestive heart failure	5 (8.1 %)	3 (6.3 %)	2 (14.3 %)	0.314
Cerebrovascular disease	4 (6.6 %)	1 (2.1 %)	3 (21.4 %)	0.035
Peripheral artery disease	5 (8.2 %)	4 (8.5 %)	1 (7.1 %)	0.678
Neoplasic disease	6 (9.8 %)	6 (12.8 %)	0 (0 %)	0.193
Malignant hemopathy	1 (1.5 %)	1 (2.0 %)	0 (0 %)	0.785
Inflammatory systemic disease	9 (14.8 %)	9 (19.1 %)	0 (0 %)	0.079
Dementia	2 (3.1 %)	2 (3.9 %)	0 (0 %)	0.613
BJI types				
Native BJI	20 (30.8 %)	16 (31.4 %)	4 (28.6 %)	0.559
Incl. arthritis	5 (25 %)	4 (25.0 %)	1 (25.0 %)	0.708
Incl. osteomyelitis	5 (25 %)	5 (31.3 %)	0 (0 %)	0.284
Incl. vertebral osteomyelitis	10 (50 %)	7 (43.8 %)	3 (75.0 %)	0.367
ODI	45 (69.2 %)	35 (68.6 %)	10 (71.4 %)	0.559
Incl. PJI	34 (75.6 %)	28 (80.0 %)	6 (60.0 %)	0.187
Incl peripheral osteosynthesis	8 (17.8 %)	6 (17.1 %)	2 (20.0 %)	0.579
Incl. vertebral osteosynthesis	3 (6.7 %)	1 (2.9 %)	2 (20.0 %)	0.119
BJI characteristics				
Evolution delay (weeks)	1.6 (0.1–6.7)	1.6 (0.4–9.2)	0.9 (0.2–3.1)	0.299
Chronic BJI (> 3 weeks)	23 (35.4 %)	19 (37.3 %)	4 (28.6 %)	0.754
Mechanism				
Hematogenous	30 (46.2 %)	24 (47.1 %)	6 (42.9 %)	1.000
Inoculation	32 (49.2 %)	25 (49.0 %)	7 (50.0 %)	1.000
Contiguity	3 (4.6 %)	2 (3.9 %)	1 (7.1 %)	0.523
MRSA	11 (16.9 %)	9 (17.6 %)	2 (14.3 %)	1.000
Plurimicrobial infection	17 (26.2 %)	16 (31.4 %)	1 (7.1 %)	0.062
Biological inflammatory syndrom	61 (95.3 %)	47 (91.0 %)	14 (100 %)	1.000
Maximal CRP value (mg/L)	164 (92–234.3)	160.2 (86.8–300.0)	264.7 (143.2–332.0)	0.245
Local and general complications				
Abscess	26 (40.0 %)	22 (43.1 %)	4 (28.6 %)	0.252
Sinus tract	23 (35.4 %)	21 (41.2 %)	2 (14.3 %)	0.056
Infective endocarditis	2 (3.7 %)	2 (4.8 %)	0 (0 %)	1.000
Hospitalization				
Length of stay (weeks)	5.6 (1.9–8.9)	5.8 (2.3–8.9)	3.8 (1.6–8.1)	0.580
Surgical management	50 (76.9 %)	38 (74.5 %)	12 (85.7 %)	0.491
Debridement (native BJI)	8 (40.0 %)	5 (31.3 %)	3 (75.0 %)	0.153
Conservative procedure^a^	24 (53.3 %)	20 (57.1 %)	4 (40.0 %)	0.274
One-stage exchange^a^	3 (6.7 %)	2 (5.7 %)	1 (10.0 %)	0.539
Two-stage exchange^a^	15 (33.3 %)	11 (31.4 %)	4 (40.0 %)	0.440
Antimicrobial therapy				
Total duration (weeks)	26.8 (17.7–42.8)	26.2 (17.9–41.6)	28.4 (17.8–48.4)	0.406
Parenteral treatment	64 (98.5 %)	50 (98.0 %)	14 (100 %)	1.000
Duration (weeks)	9.4 (5.9–24.4)	9.4 (6.3–25.1)	10.4 (4.4–16.1)	0.790
Combination therapy	65 (100 %)	51 (100 %)	14 (100 %)	NC
Duration (weeks)	25.7 (16.4–45.1)	25.6 (15.9–44.3)	27.6 (21.3–43.2)	0.442
Teicoplanin use				
IV route	51 (78.5 %)	NA	NA	NA
Loading dose	55 (85.9 %)	44 588.0 %)	11 (78.6 %)	0.521
Loading dose (mg/kg/12 h)	5.7 (4.7–6.5)	5.6 (4.7–6.5)	6.0 (5.4–6.7)	0.218
Number of injections	5 (5–5)	5 (5–5)	5 (5–5)	
Maintenance dose (mg/kg/24 h)	5.7 (4.7–6.5)	5.6 (4.7–6.5)	5.9 (5.1–6.8)	0.406
Administration route switch	7 (10.8 %)	7 (13.7 %)	0 (0 %)	0.164
Duration of treatment				
Total duration (weeks)	6.0 (2.7–9.9)	6.0 (2.9–9.7)	5.8 (3.0–11.6	0.750
IV treatment duration (weeks)	5.0 (2.9–9.7)	5.0 (3.0–9.7)	NA	NA
SC treatment duration (weeks)	6.2 (3.9–21.4)	NA	5.3 (2.8–11.6)	NA
Pharmacological data				
Number of dosages	2.5 (2–3.3)	3 (2–3)	2 (2–3.8)	0.891
Initial C_min_ (day 3 to 5, mg/L)	11.7 (9.2–16.3)	10.8 (8.6–15.2)	13.8 (11.4–16.2)	0.130
Initial C_min_ >25 mg/L	0 (0 %)	0 (0 %)	0 (0 %)	NC
Initial C_min_ <15 mg/L	36 (73.5 %)	29 (74.4 %)	7 (70.0 %)	1.000
Overdose (day 1 to 14)	10 (15.6 %)	8 (16.0 %)	2 (14.3 %)	1.000
Delay for C_min_ > 15 mg/L (days)	8.5 (6–13)	9 (6–13)	7 (4.5–9.5)	0.259
Companion drugs				
Rifampicin	16 (24.6 %)	12 (23.5 %)	4 (28.6 %)	0.732
Fluoroquinolone	29 (44.6 %)	20 (39.2 %)	9 (64.3 %)	0.131
Pristinamycin	11 (16.9 %)	11 (21.6 %)	0 (0 %)	0.102
Teicoplanin-related AE	6 (10 %)	4 (8.7 %)	2 (14.3 %)	0.617
Follow-up and outcome				
Follow-up period (weeks)	91.1 (50.6–182.6)	98.0 (58.3–194.9)	68.2 (40.7–100.3)	0.112
One-month CRP level < 10 mg/L	17 (27.9 %)	14 (28.0 %)	3 (27.3 %)	1.000
Treatment failure	27 (41.5 %)	21 (41.2 %)	6 (42.9 %)	1.000
Persisting infection	18 (28.6 %)	14 (28.0 %)	4 (30.8 %)	1.000
Relapse	6 (9.7 %)	6 (12.2 %)	0 (0 %)	0.328
Iterative surgery	23 (35.9 %)	19 (38.0 %)	4 (28.6 %)	0.754
BJI-related death	1 (1.6 %)	1 (2.0 %)	0 (0 %)	1.000
Superinfection	13 (20.0 %)	10 (19.6 %)	3 (21.4 %)	1.000

*AE* adverse event, *BJI* bone and joint infection, *BMI* body mass index, *CCI* Charlson’s comorbidity index, *C*_*min*_ plasmatic teicoplanin trough concentration, *CRP* C-reactive protein, *Incl* Including, *IV* Intravenous, *MRSA* Methicillin-resistant Staphylococcus aureus, *ODI* orthopedic device-associated infection, *PJI* prosthetic joint infection, *SC* subcutaneous

^a^ for orthopedic device-related infections

### Teicoplanin prescription modalities

Teicoplanin was used at the median dose of 5.7 (IQR, 4.7–6.5) mg/kg administrated in a single daily injection, after a loading dose (*n* = 55, 85.9 %) of 5 (IQR, 5–5) injections of 5.7 (IQR, 4.7–6.5) mg/kg/12 h. Median total duration of teicoplanin therapy was 6.0 (IQR, 2.7–9.9) weeks. Teicoplanin was mostly administrated *via* IV route (*n* = 51, 78.5 %), but 14 (21.5 %) cases were treated by SC route with no difference regarding prescription modalities (median dose, loading dose, duration) compared with IV-treated patients (Table 1). The median number of SC injections per patient was 39.5 (IQR, 24.0–86.5), with a maximum of 600 mg of teicoplanin per injection. Seven switches in administration route were observed, all in patients initially receiving IV treatment. The main teicoplanin companion drugs were fluoroquinolones (44.6 %), rifampicin (24.6 %) and pristinamycin (16.9 %).

### Pharmacological data

**Fig. 1:**
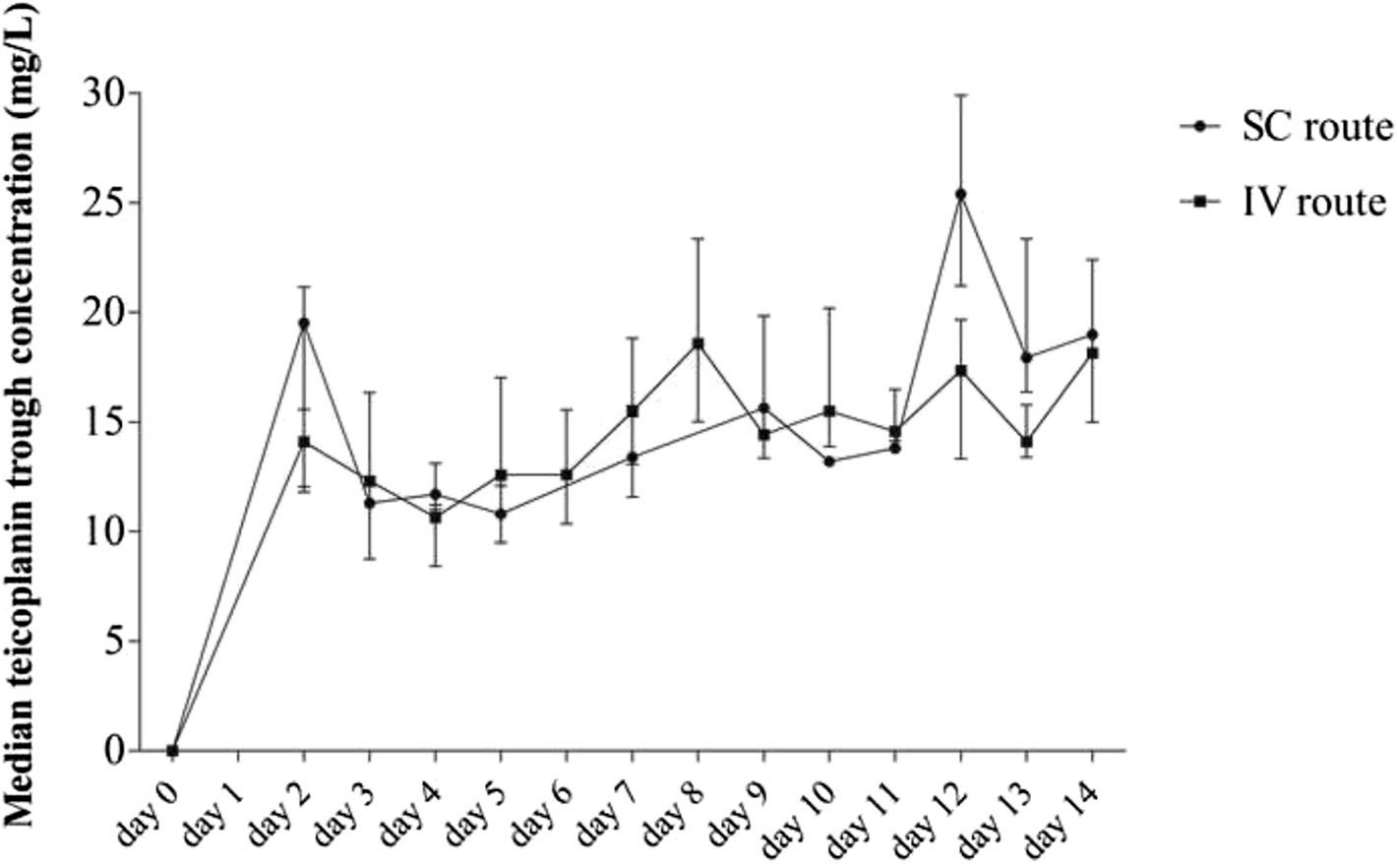
Comparison of median teicoplanin trough concentrations in intravenously- and subcutaneously-treated patients during the first 2 weeks of treatment. Data are presented as median and interquartile ranges of teicoplanin trough levels available each day after treatment initiation

During the first 14 days of treatment, at least one C_min_ value was available in 59 patients, in whom a median of 2 (IQR, 2─3) dosages was performed during this period. An early C_min_ (on day 3, 4 or 5) was available in 44 patients and was under the therapeutic target of 15 mg/L in 73.5 % of them, with a median value of 11.7 mg/L. A C_min_ >15 mg/L was finally obtained in only 39 (66.1 %) patients within the first 2 weeks of treatment, in a median delay of 9 (IQR, 6─13) days, without difference between the IV and SC routes of administration (Fig. 1). No difference was observed between patients who reached or not the therapeutic target of 15 mg/L (Additional file 1: Table S1).

During the first 2 weeks of treatment, an overexposure was observed in 8 patients who had a significantly older age (76.0 year-old, *p* = 0.007) and modified Charlson comorbidity index (7.5, *p* = 0.001) than those with no overexposure (Additional file 1: Table S1). Their baseline renal function was more frequently impaired (50.0 %, *p* = 0.049).

### Tolerance

Seven adverse events occurred in 6 (10.0 %) of the 60 included patients, consisting in 5 cutaneous rashes, 1 episode of headache, and one pancytopenia (Table 2). IV and SC routes were implicated in four and one cases, respectively (*p* = 0.617). No difference was shown between patients presenting or not a teicoplanin-related AE, and no predictive factor could be highlighted and especially chronic renal failure, daily dose and early overdose (Additional file 2: Table S2). Of note, no severe AE was reported at the injection site in the SC group. The occurrence of an adverse event did not significantly impact outcome.

**Table 2:** Description of the seven teicoplanin-related adverse events observed in 6 of the 60 included patients

Case	Modified CCI	BJI type	AE subtype	CTCAE grade	Route	Dosage	Delay	Companion drug	Stop	Hospitalization (duration)	Resolution
1	5	Acute osteomyelitis	Rash maculo-papular	2	IV	12 mg/L	7 days	None	Yes	Yes (3 days)	Yes
2	4	Acute PJI	Rash maculo-papular	2	IV	No	10 days	Oxacillin Clindamycin	Yes	No (17 days)	Yes
3	0	Acute VO	Rash maculo-papular Pancytopenia	3	SC	No	11 days	Rifampicin	Yes	No	Yes
4	5	Chronic osteomyelitis	Headache	1	IV	27.8 mg/L	20 days	Rifampicin	Yes	No	Yes
5	2	Chronic VO	Rash maculo-papular	3	SC	No	22 days	Ofloxacin	Yes	Yes (4 days)	Yes
6	2	Acute VO	Rash maculo-papular	2	IV	No	14 days	Ofloxacin	Yes	No	Yes

*AE* adverse event, *BJI* bone and joint infection, *CCI* Charlson’s comorbidity index, *CTCAE* common terminology criteria for adverse events, *IV* Intravenous, *PJI* prosthetic joint infection, *SC* subcutaneous, *VO* vertebral osteomyelitis

### Outcome

In a median follow-up of 91.1 (IQR, 50.6–182.6) weeks, 27 treatment failure were observed, including persisting infections (*n* = 18; 66.7 %), relapses (*n* = 6; 22.2 %) and/or superinfections (*n* = 13; 48.1 %), leading to iterative surgical procedure(s) in 23 (35.4 %) cases including two limb amputations. One sepsis-related death was observed.

In univariate analysis, pertinent variables associated with therapeutic failure with a *p*-value <0.15 were inflammatory systemic disease (OR, 5.600; 95 % CI, 1.056–29.683), diabetes mellitus (OR, 5.143; 95 % CI, 0.951–27.826), and abscess (OR, 4.073; 95 % CI, 1.420–11.684). The return to baseline C-reactive protein value at 1 month was associated with a lower risk of treatment failure (OR, 0.214; 95 % CI, 0.051–0.852). In multivariate analysis, in situ abscess was independently associated with treatment failure (OR, 3.641; 95 % CI, 1.110–11.944) (Additional file 3: Table S3). Of note, teicoplanin administration route did not influence the outcome.

## Discussion

Although teicoplanin is among the drugs of choice for the treatment of staphylococcal BJI, efficacy, safety and pharmacokinetics data in that specific setting are scarce. Thus, the present study provides relevant features with regards to staphylococcal BJI management. Our study is subjected to limitations BJI studies generally encounter such as the retrospective design coupled to the inherent lack of control patients. The limited patients’ recruitment, the variety of infection types, surgical management and medical treatment approaches also constitute a limitation to generalisation.

These current difficulties in the field of BJI explain the limited and controversial data available on the efficacy of teicoplanin in staphylococcal BJI. In past studies, treatment success rate ranged from 53 to 91 % [11–14]. The low success rate observed in our study (60 %) may be explained by several factors. First, there is a significant selection bias as patients were recruited in a reference center dedicated to manage complex BJI with a high-risk of failure. In addition, most of past studies included native BJI with a relatively short follow-up (<1 year). Finally, pharmacodynamics parameters may impact the outcome [15, 16]. In our study, a C_min_ reaching the therapeutic target of 15 mg/L was achieved in a quarter of cases at the first measurement (day 3 to 5) and in two thirds of patients within 2 weeks of treatment. The use of higher doses may improve these pharmacological parameters. In the study by LeFrock et al, the teicoplanin C_min_ averaged 10 mg/L after 6 days in patients receiving 6 mg/kg/day after 5 loading doses of 6 mg/kg/12 h compared to 20 mg/L from the third day in patients receiving 12 mg/kg/day after 5 loading doses of 12 mg/kg/12 h [12]. If no difference was observed regarding osteomyelitis outcome, higher doses were associated with a better outcome among patients with native septic arthritis. Nevertheless, clinical outcome according to C_min_ was not an intended end-point in the study. Greenberg et al reported a favorable outcome in patients with a C_min_ > 30 mg/L, but with no comparative data [17]. It is our belief that the loading dose should be increased to 8 mg/kg/12 h to optimize trough concentrations, particularly in case when orthopedic implant is retained. Other determinants of therapeutic success had already been described, such as inflammatory systemic disease, diabetes and abscess [18, 19]. Conversely, our study was not associated with MRSA as a negative prognostic factor as found elsewhere [20]. This last prognostic factor probably relies on the benefit of receiving anti-staphylococcal penicillins for a MSSA compared to glycopeptides [21, 22], which could not be highlighted in our series as all patients received teicoplanin, including those with MSSA infection. Finally, although all *S. aureus* isolates included in our study were tested susceptible to teicoplanin [23], the exact MIC of each isolate was not available and could consequently not be challenged as an outcome predictor. As described with vancomycin, high teicoplanin MICs (i.e., > 1.5 mg/L) have been associated with unfavorable outcome and higher mortality rate among teicoplanin-treated MRSA bacteremia [24].

Regarding safety data, our results highlighted an excellent tolerance of teicoplanin with a 10 % incidence of AE, which is consistent with the toxicity rate of 9 to 18 % observed in other similar studies [11, 13, 25]. However, the incidence of AE was probably been underestimated due to the retrospective nature of our study (memory bias for non-severe AE). Indeed, in the prospective study of LeFrock et al, the rate of AE was 35 %, requiring discontinuation of treatment in 17 % of the cases [12]. Very few data support enhanced AE related to teicoplanin dose increase [26]. LeFrock et *al* reported fever in 5.6 and 13.1 % of patients receiving 6 and 12 mg/kg/day of teicoplanin, respectively, with similar data regarding cutaneous rashes (7.6 and 15.4 %, respectively) [12]. In our study, teicoplanin daily dose and overexposure within 2 weeks of treatment were not predictors of AE. In two other studies, a dose increase from 400 to 600 mg/day was not associated with an increased risk of toxicity [27, 28].

The description of subcutaneous administration of teicoplanin is another important highlight of our study, showing similar efficacy, safety and pharmacokinetics characteristics compared to IV administration. The retrospective design may result in underestimating non-serious AE such as injection site reactions. In a recent prospective evaluation of SC teicoplanin in 30 patients, 90 % of patients presented moderate local AE (grades 1–2) and no severe local reaction (grade ≥3) [29]. Of note, none of our patients had SC teicoplanin infusion exceeding 600 mg, reported as an independent risk factor for local reaction in the study by El Samad et al [29]. Subcutaneous teicoplanin may be particularly useful in patients with BJI eligible for OPAT achieving efficacy and allowing cost reduction [30, 31]. Some authors have even proposed a 3-injections weekly regimen with a satisfactory success rate and an estimated saving of $60,000 per episode of BJI [32, 33]. However, a study has tempered this suggestion by showing a non-significant trend toward a higher risk of failure in patients treated by teicoplanin for BJI [34]. Further studies, optimally with a prospective and controlled design, are warranted to assess both the risk-benefit as well as the cost-benefit of teicoplanin in staphylococcal BJI.

## Conclusion

At the dose of 6 mg/kg/24 h after a loading dose of 5 injections of 6 mg/kg/12 h, teicoplanin appeared as a well-tolerated option in the treatment of *S. aureus* BJI, and may be recommended as an alternative to vancomycin in patients with MRSA infection or with intolerance to betalactam antibiotics. The use of higher doses must be discussed in order to optimize pharmacokinetic parameters of which clinical pertinence remains to be demonstrated. However, we believe that the loading dose should be increased to more rapidly reach the therapeutic target, which can be crucial for outcome of acute ODI with implant retention. Furthermore, subcutaneous administration of teicoplanin showed similar results in terms of efficacy, tolerance and pharmacokinetics compared to the intravenous administration, which encourage its use in OPAT. However, the implication of a multidisciplinary referral center for the management of complex BJI is needed to ensure a successful outpatient management, as suggested by the need for a close clinical, biological and pharmacological monitoring, particularly during the first 2 weeks of treatment when the majority of side effects occur.

## Additional files

Additional file 1: Table S1. Comparison of patients presenting or not a teicoplanin overdose (teicoplanin plasmatic trough concentration > 25 mg/L) and reaching or not the therapeutic concentration of 15 mg/L during the first 14 days of treatment (DOCX 19 kb)
Additional file 2: Table S2. Adverse events determinants in the 60 included patients treated by teicoplanin for a *Staphylococcus aureus* bone and joint infection (DOCX 22 kb)
Additional file 3: Table S3. Treatment failure determinants of the 65 included episodes of *Staphylococcus aureus* bone and joint infection (DOCX 25 kb)

## Abbreviations

95%CI: 95 % confidence interval
AE: Adverse event
BJI: Bone and joint infection
BMI: Body mass index
CCI: Charlson’s comorbidity index
Cmin: Trough concentration
CNS: Coagulase negative *Staphylococci*
CRP: C-reactive protein
CTCAE: Common terminology criteria for adverse events
IQR: Interquartile range
IV: Intravenous
MRSA: Methicillin-resistant *Staphylococcus aureus*
MSSA: Methicillin-susceptible *Staphylococcus aureus*
ODI: Orthopaedic device-related infection
OPAT: Outpatient parenteral antimicrobial therapy
OR: Odd ratio
PJI: Prosthetic joint infection
SC: Subcutaneous
VO: Vertebral osteomyelitis
