# Supplementary material for: Teicoplanin-based antimicrobial therapy in Staphylococcus aureus bone and joint infection: tolerance, efficacy and experience with subcutaneous administration
Source: BMC Infect Dis. 2016 Nov 3;16:622. doi: 10.1186/s12879-016-1955-7 (PMC5093939; doi:10.1186/s12879-016-1955-7)
Supplement: Additional file 1: Table S1. — Comparison of patients presenting or not a teicoplanin overdose (teicoplanin plasmatic trough concentration > 25 mg/L) and reaching or not the therapeutic concentration of 15 mg/L during the first 14 days of treatment (DOCX 19 kb) [file 12879_2016_1955_MOESM1_ESM.docx]

**Additional file 1: Table S1 – Comparison of patients presenting or not a teicoplanin overdose (teicoplanin plasmatic trough concentration > 25 mg/L) and reaching or not the therapeutic concentration of 15 mg/L during the first 14 days of treatment**

|  | | | | **Total population**  **(n=60)** | **Overdose**  **(n=8)** | ***p*-value** | **C_min_ < 15 mg/L**  **(n=20)** | ***p*-value** |
| --- | --- | --- | --- | --- | --- | --- | --- | --- |
| **Demographics** | | | |  |  |  |  |  |
|  | Sex (male) | | | 34 (56.7%) | 4 (50.0%) | 0.722 | 9 (45.0%) | 0.274 |
|  | Age (year-old) | | | 61.7 (47.5-74.7) | 76.0 (71.9-83.5) | 0.007 | 59.7 (38.5-74.3) | 0.345 |
| **Comorbidities** | | | |  |  |  |  |  |
|  | Modified CCI | | | 2.5 (1-5) | 7.5 (5.8-8.3) | 0.001 | 2 (0-4.3) | 0.119 |
|  | BMI (kg/m²) | | | 26.4 (21.4-28.7) | 28.0 (26.8-36.3) | 0.178 | 24.1 (21.1-29.1) | 0.484 |
|  | Obesity (BMI > 30) | | | 9 (15.5%) | 2 (33.3%) | 0.237 | 2 (10.0%) | 0.471 |
|  | Diabetes | | | 8 (13.3%) | 2 (25.0%) | 0.295 | 2 (10.0%) | 0.704 |
|  | Immunosuppression | | | 10 (16.7%) | 2 (25.0%) | 0.613 | 3 (15.0%) | 1.000 |
|  | Chronic renal failure | | | 9 (16.1%) | 3 (50.0%) | 0.049 | 3 (15.8%° | 1.000 |
|  | Chronic hepatic disease | | | 2 (3.6%) | 0 (0%) | 1.000 | 1 (5.3%) | 1.000 |
|  | Chronic pulmonary disease | | | 12 (21.4%) | 1 (16.7%) | 1.000 | 3 (15.8%) | 0.730 |
|  | Congestive heart failure | | | 5 (8.8%) | 2 (28.6%) | 0.113 | 1 (5.3%) | 0.652 |
|  | Cerebrovascular disease | | | 4 (7.1%) | 2 (33.3%) | 0.055 | 0 (0%) | 0.286 |
|  | Peripheral artery disease | | | 5 (8.9%) | 0 (0%) | 1.000 | 0 (0%) | 0.286 |
|  | Neoplasic disease | | | 6 (10.7%) | 2 (33.3%) | 0.123 | 2 (10.5%) | 1.000 |
|  | Malignant hemopathy | | | 1 (1.7%) | 0 (0%) | 1.000 | 0 (0%) | 1.000 |
|  | Inflammatory systemic disease | | | 8 (14.3%) | 0 (0%) | 1.000 | 2 (10.5%) | 1.000 |
|  | Dementia | | | 2 (3.3%) | 1 (12.5%) | 0.255 | 1 (5.0%) | 1.000 |
| **Teicoplanin use** | | | |  |  |  |  |  |
|  | IV route | | | 46 (76.7%) | 6 (75.0%) | 1.000 | 16 (80.0%) | 0.753 |
|  | Loading dose | | | 50 (84.7%) | 6 (85.7%) | 1.000 | 17 (85.0%) | 1.000 |
|  |  | Loading dose (mg/kg/12h) | | 5.8 (4.9-6.5) | 6.0 (6.0-6.3) | 0.491 | 5.7 (5.1-6.5) | 0.641 |
|  |  | Number of injections | | 5 (5-5) | 5 (5-5) | 0.648 | 5 (5-5) | 0.684 |
|  | Maintenance dose (mg/kg/24h) | | | 5.8 (4.8-6.6) | 6.2 (6.0-6.9) | 0.231 | 5.3 (4.6-6.2) | 0.128 |
|  | Administration route switch | | | 5 (8.3%) | 1 (12.5%) | 0.531 | 0 (0%) | 0.156 |
|  | Treatment duration | | |  |  |  |  |  |
|  |  | Total duration (weeks) | | 5.4 (2.7-9.8) | 8.9 (3.3-25.9) | NA | 3.1 (2.0-6.3) | NA |
|  |  | IV treatment duration (weeks) | | 4.6 (2.9-8.4) | 4.6 (3.1-15.8) | NA | 3.9 (2.1-7.1) | NA |
|  |  | SC treatment duration (weeks) | | 5.3 (3.4-12.3) | 24.1 (18.1-45.1) | NA | 2.0 (1.4-2.8) | NA |
|  | Pharmacological data | | |  |  |  |  |  |
|  |  | Number of dosages | | 2 (2-3) | 4 (3-5) | 0.002 | 2 (1.8-2.0) | <10^-3^ |
|  |  | Initial C_min_ (day 3 to 5, mg/L) | | 12.4 (10.2-16.3) | 11.2 (8.9-13.0) | 0.413 | 10.7 (9.4-11.2) | 0.010 |
|  |  |  | Initial C_min_ >25 mg/L | 0 (0%) | 0 (0%) | NC | 0 (0%) | NA |
|  |  |  | Initial C_min_ <15 mg/L | 32 (72.7%) | 6 (85.7%) | 0.653 | 14 (100%) | NA |
|  |  | Overdose (day 1 to 14) | | 8 (13.6%) | NA | NA | 0 (0%) | NA |
|  |  | Delay for C_min_ >15 mg/L (days) | | 9 (6-13) | 9 (7.8-12.3) | 0.674 | NA | NA |
|  | Companion drug | | |  |  |  |  |  |
|  |  | Rifampicin | | 16 (26.7%) | 2 (25.0%) | 1.000 | 7 (35.0%) | 0.366 |
|  |  | Fluoroquinolone | | 27 (45.0%) | 5 (62.5%) | 0.450 | 6 (30.0%) | 0.103 |
|  |  | Pristinamycin | | 9 (15.0%) | 0 (0%) | 0.582 | 3 (15.0%) | 1.000 |
| **Teicoplanin-related AE** | | | | 6 (10.0%) | 2 (25.0%) | 0.183 | 3 (15.0%) | 0.398 |
| **Follow-up and outcome** | | | |  |  |  |  |  |
|  | Follow-up period (weeks) | | | 91.9 (54.6-192.3) | 96.5 (52.8-151.3) | 0.713 | 86.9 (61.8-271.6) | 0.498 |
|  | One-month CRP level < 10 mg/L | | | 17 (30.4%) | 0 (0%) | 0.048 | 7 (38.9%) | 0.535 |
|  | Treatment failure | | | 25 (41.7%) | 3 (37.5%) | 1.000 | 6 (30.0%) | 0.273 |
|  |  | Persisting infection | | 18 (31.0%) | 2 (25.0%) | 1.000 | 4 (22.2%) | 0.537 |
|  |  | Relapse | | 6 (10.5%) | 1 (12.5%) | 0.552 | 1 (5.6%) | 1.000 |
|  |  | Iterative surgery | | 21 (35.6%) | 1 (12.5%) | 0.241 | 6 (31.6%) | 1.000 |
|  |  | BJI-related death | | 1 (1.7%) | 0 (0%) | 1.000 | 0 (0%) | 1.000 |
|  |  | Superinfection | | 11 (18.3%) | 1 (12.5%) | 1.000 | 1 (5.0%) | 0.079 |
| **Initial hospitalization (weeks)** | | | | 5.4 (1.6-7.5) | 9.2 (4.8-13.6) | 0.331 | 5.6 (2.3-7.2) | 0.784 |

*AE, Adverse event; BJI, Bone and joint infection; BMI, Body mass index; CCI, Charlson’s comorbidity index; C_min_, Plasmatic teicoplanin trough concentration; CRP, C-reactive protein; IV, Intravenous; SC, Subcutaneous.*
