# Supplementary material for: Teicoplanin-based antimicrobial therapy in Staphylococcus aureus bone and joint infection: tolerance, efficacy and experience with subcutaneous administration
Source: BMC Infect Dis. 2016 Nov 3;16:622. doi: 10.1186/s12879-016-1955-7 (PMC5093939; doi:10.1186/s12879-016-1955-7)
Supplement: Additional file 2: Table S2. — Adverse events determinants in the 60 included patients treated by teicoplanin for a Staphylococcus aureus bone and joint infection (DOCX 22 kb) [file 12879_2016_1955_MOESM2_ESM.docx]

**Additional file 2: Table S2 – Adverse events determinants in the 60 included patients treated by teicoplanin for a *Staphylococcus aureus* bone and joint infection**

|  | | | | **Total population**  **(n=60)** | **Adverse event**  **(n=6)** | **No adverse event**  **(n=54)** | ***p*-value** | **OR (95%CI)** | ***p*-value** |
| --- | --- | --- | --- | --- | --- | --- | --- | --- | --- |
| **Demographics** | | | |  |  |  |  |  |  |
|  | Sex (male) | | | 34 (56.7%) | 2 (33.3%) | 32 (59.3%) | 0.388 | 0.344 (0.058-2.043) | 0.240 |
|  | Age (year-old) | | | 61.7 (47.5-74.7) | 61.6 (57.9-71.7) | 61.8 (47.2-74.5) | 0.824 | 1.017 (0.650-1.591)^a^ | 0.942 |
| **Comorbidities** | | | |  |  |  |  |  |  |
|  | Modified CCI | | | 2.5 (1-5) | 3 (2-4.8) | 2.5 (1.0-5.0) | 1.000 | 0.952 (0.709-1.279) | 0.744 |
|  | BMI (kg/m²) | | | 26.4 (21.4-28.7) | 21.9 (20.8-29.2) | 26.6 (21.5-28.6) | 0.542 | 0.947 (0.809-1.108) | 0.495 |
|  | Obesity (BMI > 30) | | | 9 (15.5%) | 2 (33.3%) | 7 (13.5%) | 0.231 | 3.214 (0.493-20.958) | 0.222 |
|  | Diabetes | | | 8 (13.3%) | 1 (16.7%) | 7 (13.0%) | 1.000 | 1.343 (0.136-13.250) | 0.801 |
|  | Immunosuppression | | | 10 (16.7%) | 1 (16.7%) | 9 (16.7%) | 1.000 | 1.000 (0.104-9.614) | 1.000 |
|  | Chronic renal failure | | | 9 (16.1%) | 2 (33.3%) | 7 (14.0%) | 0.244 | 3.071 (0.471-20.047) | 0.241 |
|  | Chronic hepatic disease | | | 2 (3.6%) | 0 (0%) | 2 (4.0%) | 1.000 | NC | NC |
|  | Chronic pulmonary disease | | | 12 (21.4%) | 1 (16.7%) | 11 (22.0%) | 1.000 | 0.709 (0.075-6.720) | 0.764 |
|  | Congestive heart failure | | | 5 (8.8%) | 0 (0%) | 5 (9.8%) | 1.000 | NC | NC |
|  | Cerebrovascular disease | | | 4 (7.1%) | 0 (0%) | 4 (8.0%) | 1.000 | NC | NC |
|  | Peripheral artery disease | | | 5 (8.9%) | 0 (0%) | 5 (10.0%) | 1.000 | NC | NC |
|  | Neoplasic disease | | | 6 (10.7%) | 0 (0%) | 6 (12.0%) | 1.000 | NC | NC |
|  | Malignant hemopathy | | | 1 (1.7%) | 0 (0%) | 1 (1.9%) | 1.000 | NC | NC |
|  | Inflammatory systemic disease | | | 8 (14.3%) | 1 (16.7%) | 7 (14.0%) | 1.000 | 1.229 (0.124-12.142) | 0.860 |
|  | Dementia | | | 2 (3.3%) | 0 (0%) | 2 (3.7%) | 1.000 | NC | NC |
| **Teicoplanin use** | | | |  |  |  |  |  |  |
|  | IV route | | | 46 (76.7%) | 4 (66.7%) | 42 (77.8%) | 0.617 | 0.571 (0.093-3.508) | 0.546 |
|  | Loading dose | | | 50 (84.7%) | 5 (83.3%) | 45 (84.9%) | 1.000 | 0.889 (0.091-8.646) | 0.919 |
|  |  | Loading dose (mg/kg/12h) | | 5.8 (4.9-6.5) | 5.7 (5.4-6.5) | 5.8 (4.9-6.5) | 0.809 | 1.850 (0.001-4228.586)^b^ | 0.876 |
|  |  | Number of injections | | 5 (5-5) | 5 (5-5) | 5 (5-5) | 0.485 | 0.870 (0.559-1.354) | 0.536 |
|  | Maintenance dose (mg/kg/24h) | | | 5.8 (4.8-6.6) | 5.7 (5.4-6.8) | 5.8 (4.8-6.5) | 0.714 | 5.150 (0.004-6359.356)^b^ | 0.652 |
|  | Administration route switch | | | 27 (50.0%) | 1 (20.0%) | 26 (53.1%) | 0.351 | 0.221 (0.023-2.124) | 0.191 |
|  | Treatment duration | | |  |  |  |  |  |  |
|  |  | Total duration (weeks) | | 5.4 (2.7-9.8) | 2.5 (1.5-3.9) | 6.0 (3.0-9.8) | 0.094 | NA | NA |
|  |  | IV treatment duration (sem) | | 4.6 (2.9-8.4) | 2.4 (1.3-7.4) | 5.0 (3.0-8.4) | 0.350 | NA | NA |
|  |  | SC treatment duration (sem) | | 5.3 (3.4-12.3) | 3.7 (2.6-13.9) | 5.9 (4.3-12.0) | 0.545 | NA | NA |
|  | Pharmacological data | | |  |  |  |  |  |  |
|  |  | Number of dosages | | 2 (2-3) | 3 (2.3-3.8) | 2 (2-3) | 0.417 | NA | NA |
|  |  | Initial C_min_ (day 3 to 5, mg/L) | | 12.4 (10.2-16.3) | 10.8 (5.4-11.3) | 12.5 (10.5-16.6) | 0.116 | 0.818 (0.656-1.020) | 0.075 |
|  |  |  | Initial C_min_ >25 mg/L | 0 (0%) | 0 (0%) | 0 (0%) | NC | NC | NC |
|  |  |  | Initial C_min_ <15 mg/L | 32 (72.7%) | 5 (83.3%) | 27 (71.1%) | 1.000 | 2.037 (0.213-19.494) | 0.537 |
|  |  | Overdose (day 1 to 14) | | 8 (13.6%) | 2 (33.3%) | 6 (11.3%) | 0.183 | 3.917 (0.587-26.139) | 0.159 |
|  |  | Delay for C_min_ > 15 mg/L (days) | | 9 (6-13) | 9 (6.5-12) | 8.5 (6-13) | 0.832 | 1.019 (0.769-1.351) | 0.894 |
|  | Companion drug | | |  |  |  |  |  |  |
|  |  | Rifampicin | | 16 (26.7%) | 2 (33.3%) | 14 (25.9%) | 0.653 | 1.429 (0.235-8.670) | 0.698 |
|  |  | Fluoroquinolone | | 27 (45.0%) | 2 (33.3%) | 25 (46.3%) | 0.681 | 0.580 (0.098-3.438) | 0.549 |
|  |  | Pristinamycin | | 9 (15.0%) | 0 (0%) | 9 (16.7%) | 0.578 | NC | NC |
| **Follow-up and outcome** | | | |  |  |  |  |  |  |
|  | Follow-up period (weeks) | | | 91.9 (54.6-192.3) | 114.1 (80.3-170.9) | 91.9 (46.6-198.9) | 0.436 | NA | NA |
|  | One-month CRP level < 10 mg/L | | | 17 (30.4%) | 1 (16.7%) | 16 (32.0%) | 0.655 | 0.425 (0.046-3.944) | 0.452 |
|  | Treatment failure | | | 25 (41.7%) | 4 (66.7%) | 21 (38.9%) | 0.223 | 3.143 (0.528-18.700) | 0.208 |
|  |  | Persisting infection | | 18 (31.0%) | 2 (40.0%) | 16 (30.2%) | 0.641 | 1.542 (0.235-10.132) | 0.652 |
|  |  | Relapse | | 6 (10.5%) | 1 (20.0%) | 5 (9.6%) | 0.439 | 2.350 (0.218-25.325) | 0.481 |
|  |  | Iterative surgery | | 21 (35.6%) | 3 (50.0%) | 18 (34.0%) | 0.656 | 1.944 (0.356-10.625) | 0.443 |
|  |  | BJI-related death | | 1 (1.7%) | 0 (0%) | 1 (1.9%) | 1.000 | NC | NC |
|  |  | Superinfection | | 11 (18.3%) | 2 (33.3%) | 8 (16.7%) | 0.302 | 2.500 (0.396-15.774) | 0.330 |
| **Initial hospitalization (weeks)** | | | | 5.4 (1.6-7.5) | 6.4 (5.6-11.1) | 5 (1.6-7.3) | 0.172 | NA | NA |

*95%CI, 95% confident interval; AE, Adverse event; BJI, Bone and joint infection; BMI, Body mass index; CCI, Charlson’s comorbidity index; C_min_, Plasmatic teicoplanin trough concentration; CRP, C-reactive protein; OR, Odd ratio; IV, Intravenous; SC, Subcutaneous.*

*^a^ for 10 additional years; ^b^ for 10 additional mg/kg.*
