# Supplementary material for: Teicoplanin-based antimicrobial therapy in Staphylococcus aureus bone and joint infection: tolerance, efficacy and experience with subcutaneous administration
Source: BMC Infect Dis. 2016 Nov 3;16:622. doi: 10.1186/s12879-016-1955-7 (PMC5093939; doi:10.1186/s12879-016-1955-7)
Supplement: Additional file 3: Table S3. — Treatment failure determinants of the 65 included episodes of Staphylococcus aureus bone and joint infection (DOCX 25 kb) [file 12879_2016_1955_MOESM3_ESM.docx]

**Additional file 3: Table S3 – Treatment failure determinants of the 65 included episodes of *Staphylococcus aureus* bone and joint infection**

|  | | | | **Total population**  **(n=65)** | **Favorable outcome**  **(n=38)** | **Treatment failure**  **(n=27)** | ***p*-value** | **OR (95%CI)** | ***p*-value** |
| --- | --- | --- | --- | --- | --- | --- | --- | --- | --- |
| **Demographics** | | | |  |  |  |  |  |  |
|  | Sex (male) | | | 34 (52.3%) | 20 (52.6%) | 14 (51.9%) | 1.000 | 0.969 (0.361-2.602) | 0.951 |
|  | Age (year-old) | | | 61.8 (49.0-74.0) | 62.8 (48.8-74.9) | 61.4 (50.0-69.3) | 0.527 | 0.933 (0.712-1.223)^a^ | 0.616 |
| **Comorbidities** | | | |  |  |  |  |  |  |
|  | Modified CCI | | | 3 (1-5) | 2.5 (1-5) | 3 (2-5) | 0.436 | 1.084 (0.915-1.284) | 0.353 |
|  | BMI (kg/m²) | | | 27.0 (21.6-29.7) | 26.9 (21.7-29.6) | 28.0 (21.3-29.9) | 0.902 | 0.994 (0.920-1.073) | 0.869 |
|  | Obesity (BMI > 30) | | | 14 (22.2%) | 7 (19.4%) | 7 (25.9%) | 0.557 | 1.450 (0.440-4.778) | 0.541 |
|  | Diabetes | | | 8 (12.3%) | 2 (5.3%) | 6 (22.2%) | 0.058 | 5.143 (0.951-27.826) | 0.057 |
|  | Immunosuppression | | | 11 (16.9%) | 6 (15.8%) | 5 (18.5%) | 1.000 | 1.212 (0.329-4.470) | 0.773 |
|  | Chronic renal failure | | | 9 (14.8%) | 3 (8.8%) | 6 (22.2%) | 0.167 | 2.952 (0.664-13.133) | 0.155 |
|  | Chronic hepatic disease | | | 2 (3.3%) | 0 (0%) | 2 (7.4%) | 0.192 | NC | NC |
|  | Chronic pulmonary disease | | | 15 (24.6%) | 7 (20.6%) | 8 (29.6%) | 0.551 | 1.624 (0.503-5.243) | 0.417 |
|  | Congestive heart failure | | | 5 (8.1%) | 3 (8.6%) | 2 (7.4%) | 1.000 | 0.853 (0.132-5.504) | 0.868 |
|  | Cerebrovascular disease | | | 4 (6.6%) | 3 (8.8%) | 1 (3.7%) | 0.623 | 0.397 (0.039-4.054) | 0.436 |
|  | Peripheral artery disease | | | 5 (8.2%) | 2 (5.9%) | 3 (11.1%) | 0.647 | 2.000 (0.310-12.923) | 0.467 |
|  | Neoplasic disease | | | 6 (9.8%) | 3 (8.8%) | 3 (11.1%) | 1.000 | 1.292 (0.239-6.977) | 0.766 |
|  | Malignant hemopathy | | | 1 (1.5%) | 1 (2.6%) | 0 (0%) | 1.000 | NC | NC |
|  | Inflammatory systemic disease | | | 9 (14.8%) | 2 (5.9%) | 7 (25.9%) | 0.065 | 5.600 (1.056-29.683) | 0.043 |
|  | Dementia | | | 2 (3.1%) | 0 (0%) | 2 (7.4%) | 0.169 | NC | NC |
| **BJI type** | | | |  |  |  |  |  |  |
|  | Native BJI | | | 20 (30.8%) | 14 (36.8%) | 6 (22.2%) | 0.278 | 0.490 (0.160-1.503) | 0.212 |
|  |  | Incl. arthritis | | 5 (25%) | 4 (28.6%) | 1 (16.7%) | 0.393 | 0.327 (0.034-3.102) | 0.330 |
|  |  | Incl. osteomyelitis | | 5 (25%) | 2 (14.3%) | 3 (50.0%) | 0.642 | 2.250 (0.349-14.486) | 0.393 |
|  |  | Incl. vertebral osteomyelitis | | 10 (50%) | 8 (57.1%) | 2 (33.3%) | 0.175 | 0.300 (0.058-1.543) | 0.150 |
|  | ODI | | | 45 (69.2%) | 24 (63.2%) | 21 (77.8%) | 0.278 | 2.042 (0.665-6.266) | 0.212 |
|  |  | Incl. PJI | | 34 (75.6%) | 20 (83.3%) | 14 (66.7%) | 0.299 | 0.400 (0.098-1.631) | 0.201 |
|  |  | Incl peripheral osteosynthesis | | 8 (17.8%) | 3 (12.5%) | 5 (23.8%) | 0.443 | 2.187 (0.454-10.538) | 0.329 |
|  |  | Incl. vertebral osteosynthesis | | 3 (6.7%) | 1 (4.2%) | 2 (9.5%) | 0.592 | 2.421 (0.204-28.800) | 0.484 |
| **BJI characteristics** | | | |  |  |  |  |  |  |
|  | Evolution delay (weeks) | | | 1.6 (0.1-6.7) | 1.9 (1.0-9.4) | 0.6 (0.1-1.9) | 0.032 | 0.993 (0.965-1.021) | 0.608 |
|  |  | Chronic BJI (> 3 weeks) | | 23 (35.4%) | 17 (44.7%) | 6 (22.2%) | 0.072 | 0.353 (0.116-1.071) | 0.066 |
|  | Mechanism | | |  |  |  |  |  |  |
|  |  | Haematogenous | | 30 (46.2%) | 18 (47.4%) | 12 (44.4%) | 1.000 | 0.889 (0.330-2.394) | 0.816 |
|  |  | Inoculation | | 32 (49.2%) | 18 (47.4%) | 14 (51.9%) | 0.804 | 1.197 (0.446-3.213) | 0.722 |
|  |  | Contiguity | | 3 (4.6%) | 2 (5.3%) | 1 (3.7%) | 1.000 | 0.692 (0.060-8.046) | 0.769 |
|  | MRSA | | | 11 (16.9%) | 10 (26.3%) | 1 (3.7%) | 0.020 | 0.108 (0.013-0.901) | 0.040 |
|  | Plurimicrobial infection | | | 17 (26.2%) | 11 (28.9%) | 6 (22.2%) | 0.582 | 0.701 (0.223-2.207) | 0.544 |
| **Biological inflammatory syndrom** | | | | 61 (95.3%) | 34 (91.9%) | 27 (100%) | 0.257 | NC | NC |
|  | Maximal CRP value (mg/L) | | | 164 (92-234.3) | 161 (86-300) | 196 (112.3-335) | 0.335 | 1.018 (0.982-1.054)^b^ | 0.329 |
| **Local and general complications** | | | |  |  |  |  |  |  |
|  | Abscess | | | 26 (40.0%) | 10 (26.3%) | 16 (59.3%) | 0.011 | 4.073 (1.420-11.684) | 0.009 |
|  | Sinus tract | | | 23 (35.4%) | 12 (31.6%) | 11 (40.7%) | 0.599 | 1.490 (0.533-4.165) | 0.447 |
|  | Infective endocarditis | | | 2 (3.7%) | 1 (3.6%) | 1 (3.8%) | 1.000 | 1.080 (0.064-18.204) | 0.957 |
| **Hospitalization** | | | |  |  |  |  |  |  |
|  | Length of stay (weeks) | | | 5.6 (1.9-8.9) | 4.3 (1.8-7.4) | 6.1 (2.0-11.1) | 0.162 | NA | NA |
| **Surgical management** | | | | 50 (76.9%) | 27 (71.1%) | 23 (85.2%) | 0.239 | 2.343 (0.656-8.361) | 0.190 |
|  | Debridement (native BJI) | | | 8 (40.0%) | 5 (35.7%) | 3 (50.0%) | 0.642 | 1.800 (0.259-12.502) | 0.552 |
|  | Conservative procedure^c^ | | | 24 (53.3%) | 11 (45.8%) | 13 (61.9%) | 0.373 | 1.920 (0.583-6.324) | 0.283 |
|  | One-stage exchange^c^ | | | 3 (6.7%) | 2 (8.3%) | 1 (4.8%) | 1.000 | 0.550 (0.046-6.539) | 0.636 |
|  | Two-stage exchange^c^ | | | 15 (33.3%) | 9 (37.5%) | 6 (28.6%) | 0.752 | 0.667 (0.190-2.343) | 0.527 |
| **Antimicrobial therapy** | | | |  |  |  |  |  |  |
|  | Total duration (weeks) | | | 26.8 (17.7-42.8) | 25.1 (14.6-31.6) | 36.9 (25.3-58.6) | 0.007 | NA | NA |
|  | Parenteral treatment | | | 64 (98.5%) | 38 (100%) | 26 (96.3%) | 0.415 | NC | NC |
|  |  | Duration (weeks) | | 9.4 (5.9-24.4) | 8.4 (5.9-13.3) | 13.9 (6.6-43.3) | 0.082 | NA | NA |
|  | Combination therapy | | | 65 (100%) | 38 (100%) | 27 (100%) | NC | NC | NC |
|  |  | Duration (weeks) | | 25.7 (16.4-45.1) | 25.6 (14.1-31.1) | 27.6 (23.8-54.5) | 0.060 | NA | NA |
| **Teicoplanin use** | | | |  |  |  |  |  |  |
|  | IV route | | | 51 (78.5%) | 30 (78.9%) | 21 (77.8%) | 1.000 | 0.933 (0.282-3.088) | 0.910 |
|  | Loading dose | | | 55 (85.9%) | 32 (86.5%) | 23 (85.2%) | 1.000 | 0.898 (0.217-3.716) | 0.882 |
|  |  | Loading dose (mg/kg/12h) | | 5.7 (4.7-6.5) | 5.7 (4.5-6.5) | 5.7 (4.9-6.5) | 0.402 | 6.392 (0.066-616.156)^d^ | 0.426 |
|  |  | Number of injections | | 5 (5-5) | 5 (5-5) | 5 (5-5) | 0.836 | 0.923 (0.684-1.245) | 0.600 |
|  | Maintenance dose (mg/kg/24h) | | | 5.7 (4.7-6.5) | 5.6 (4.1-6.4) | 5.9 (5.0-6.7) | 0.165 | 23.756 (0.415-1359.969)^d^ | 0.125 |
|  | Administration route switch | | | 7 (10.8%) | 3 (7.9%) | 4 (14.8%) | 0.437 | 2.029 (0.415-9.917) | 0.382 |
|  | Duration of treatment | | |  |  |  |  |  |  |
|  |  | Total duration (weeks) | | 6.0 (2.7-9.9) | 6.1 (3.2-9.7) | 4.6 (2.6-18.8) | 0.968 | NA | NA |
|  |  | IV treatment duration (weeks) | | 5.0 (2.9-9.7) | 5.9 (2.7-9.7) | 4.4 (3.0-13.7) | 0.933 | NA | NA |
|  |  | SC treatment duration (weeks) | | 6.2 (3.9-21.4) | 5.9 (4.3-12.0) | 10.6 (2.4-24.1) | 0.565 | NA | NA |
|  | Pharmacological data | | |  |  |  |  |  |  |
|  |  | Number of dosages | | 2.5 (2-3.3) | 2 (2-3) | 3 (2-3.8) | 0.605 | NA | NA |
|  |  | Initial C_min_ (day 3 to 5, mg/L) | | 11.7 (9.2-16.3) | 11.3 (8.5-15.8) | 12.3 (10.0-15.4) | 0.845 | 0.992 (0.882-1.117) | 0.900 |
|  |  |  | Initial C_min_ >25 mg/L | 0 (0%) | 0 (0%) | 0 (0%) | NC | NC | NC |
|  |  |  | Initial C_min_ <15 mg/L | 36 (73.5%) | 22 (73.3%) | 14 (73.7%) | 1.000 | 1.018 (0.277-3.747) | 0.978 |
|  |  | Overdose (day 1 to 14) | | 10 (15.6%) | 5 (13.2%) | 5 (19.2%) | 0.728 | 1.517 (0.405-6.092) | 0.513 |
|  |  | Delay for C_min_ > 15 mg/L (days) | | 8.5 (6-13) | 7 (6-11.5) | 9 (6.8-14) | 0.296 | 1.074 (0.925-1.246) | 0.350 |
|  | Companion drug | | |  |  |  |  |  |  |
|  |  | Rifampicin | | 16 (24.6%) | 10 (26.3%) | 6 (22.2%) | 0.777 | 0.800 (0.251-2.551) | 0.706 |
|  |  | Fluoroquinolone | | 29 (44.6%) | 17 (44.7%) | 12 (44.4%) | 1.000 | 0.988 (0.366-2.666) | 0.981 |
|  |  | Pristinamycin | | 11 (16.9%) | 7 (18.4%) | 4 (14.8%) | 0.751 | 0.770 (0.201-2.946) | 0.703 |
| **Follow-up and outcome** | | | |  |  |  |  |  |  |
|  | Follow-up period (weeks) | | | 91.1 (50.6-182.6) | 72.6 (44.7-106.9) | 171.1 (81.8-246.6) | 0.009 | NA | NA |
|  | One-month CRP level < 10 mg/L | | | 17 (27.9%) | 14 (38.9%) | 3 (12.0%) | 0.040 | 0.214 (0.054-0.852) | 0.029 |
|  |  | Persisting infection | | 18 (28.6%) | NA | NA | NA | NA | NA |
|  |  | Relapse | | 6 (9.7%) | NA | NA | NA | NA | NA |
|  |  | Iterative surgery | | 23 (35.9%) | NA | NA | NA | NA | NA |
|  |  | BJI-related death | | 1 (1.6%) | NA | NA | NA | NA | NA |
|  |  | Superinfection | | 13 (20.0%) | NA | NA | NA | NA | NA |
|  | Functional sequel | | | 33 (57.9%) | 14 (41.2%) | 19 (82.6%) | 0.003 | NA | NA |

*AE, Adverse event; BJI, Bone and joint infection; BMI, Body mass index; CCI, Charlson’s comorbidity index; C_min_, Plasmatic teicoplanin trough concentration; CRP, C-reactive protein; Incl, Including; IV, Intravenous; MRSA, Methicillin-resistant Staphylococcus aureus; ODI, Orthopedic device-associated infection; PJI, Prosthetic joint infection; SC, Subcutaneous.*

*^a^ for 10 additional years; ^b^ for 10 additional mg/L;* ^c^ *for orthopedic device-related infections; ^d^ for 10 additional mg/kg.*
